# Biomarkers of Environmental Enteropathy are Positively Associated with Immune Responses to an Oral Cholera Vaccine in Bangladeshi Children

**DOI:** 10.1371/journal.pntd.0005039

**Published:** 2016-11-08

**Authors:** Muhammad Ikhtear Uddin, Shahidul Islam, Naoshin S. Nishat, Motaher Hossain, Tanzeem Ahmed Rafique, Rasheduzzaman Rashu, Mohammad Rubel Hoq, Yue Zhang, Amit Saha, Jason B. Harris, Stephen B. Calderwood, Taufiqur Rahman Bhuiyan, Edward T. Ryan, Daniel T. Leung, Firdausi Qadri

**Affiliations:** 1 Mucosal Immunology and Vaccinology Laboratory, Infectious Diseases Division, icddr,b, Dhaka, Bangladesh; 2 Department of Internal Medicine, Division of Epidemiology, University of Utah, School of Medicine, Salt Lake City, Utah, United States of America; 3 Department of Internal Medicine, Division of Infectious Diseases, University of Utah, School of Medicine, Salt Lake City, Utah, United States of America; 4 Department of Pathology, Division of Microbiology and Immunology, University of Utah, School of Medicine, Salt Lake City, Utah, United States of America; 5 Department of Immunology and Infectious Diseases, Division of Infectious Diseases, Massachusetts General Hospital, Boston, Massachusetts, United States of America; 6 Department of Pediatrics, Harvard Medical School, Boston, Massachusetts, United States of America; 7 Department of Medicine, Harvard Medical School, Boston, Massachusetts, United States of America; Instituto Butantan, BRAZIL

## Abstract

Environmental enteropathy (EE) is a poorly understood condition that refers to chronic alterations in intestinal permeability, absorption, and inflammation, which mainly affects young children in resource-limited settings. Recently, EE has been linked to suboptimal oral vaccine responses in children, although immunological mechanisms are poorly defined. The objective of this study was to determine host factors associated with immune responses to an oral cholera vaccine (OCV). We measured antibody and memory T cell immune responses to cholera antigens, micronutrient markers in blood, and EE markers in blood and stool from 40 Bangladeshi children aged 3–14 years who received two doses of OCV given 14 days apart. EE markers included stool myeloperoxidase (MPO) and alpha anti-trypsin (AAT), and plasma endotoxin core antibody (EndoCab), intestinal fatty acid binding protein (i-FABP), and soluble CD14 (sCD14). We used multiple linear regression analysis with LASSO regularization to identify host factors, including EE markers, micronutrient (nutritional) status, age, and HAZ score, predictive for each response of interest. We found stool MPO to be positively associated with IgG antibody responses to the B subunit of cholera toxin (P = 0.03) and IgA responses to LPS (P = 0.02); plasma sCD14 to be positively associated with LPS IgG responses (P = 0.07); plasma i-FABP to be positively associated with LPS IgG responses (P = 0.01) and with memory T cell responses specific to cholera toxin (P = 0.01); stool AAT to be negatively associated with IL-10 (regulatory) T cell responses specific to cholera toxin (P = 0.02), and plasma EndoCab to be negatively associated with cholera toxin-specific memory T cell responses (P = 0.02). In summary, in a cohort of children 3–14 years old, we demonstrated that the majority of biomarkers of environmental enteropathy were positively associated with immune responses after vaccination with an OCV.

## Author Summary

Cholera is a life-threatening diarrheal disease that affects millions of people worldwide. Currently available oral cholera vaccines are less effective in young children, and some have hypothesized that this is related to environmental enteropathy, a problem in the gut characterized by alterations in intestinal permeability, absorption, and inflammation, which mainly affects young children in resource-limited settings. We measured cholera-specific immune responses in 40 Bangladeshi children aged 3–14 who received an oral cholera vaccine. We then identified host factors, such as enteropathy biomarkers, sex, age, and micronutrient status, associated with each immune response. Unexpectedly, we found enteropathy biomarkers to be positively associated with immune responses to vaccine, underlining the complexity of the interaction between enteropathy and oral vaccine immunogenicity.

## Introduction

The immunogenicity and efficacy of oral vaccines are lower in developing countries compared to those in developed countries. This hypo-responsiveness is predominantly seen in lower age group children [1,2,3,4]. Environmental enteropathy (EE) is an acquired syndrome, characterized by villous blunting, crypt hyperplasia, and increased intraepithelial lymphocytes and pro-inflammatory cytokine responses [5,6]. EE is known to be common in settings with poor water, sanitation and hygiene infrastructure such as in low- and lower-middle income countries. It is hypothesized that repeated enteric infections are the underlying cause of this subclinical condition, which also result in reduced efficacy of vaccines. However, the mechanisms underlying this entity are not well understood [7,8,9]

Cholera is a dehydrating diarrheal disease endemic in more than 50 countries across the world. It is caused by infection with *Vibrio cholerae*, present in contaminated water and food [10]. The disease burden is highest in children under 5 years of age [11]. However, currently available and WHO recommended oral cholera vaccines (OCV) show less pronounced immune responses and protection in this age group of children compared with older children and adults [1,12,13]. Little is known regarding how host factors, such as malnutrition, micronutrient deficiency, and enteropathy, affect vaccine-induced immune responses in children. The objective of this study was to determine how host factors impact immune responses in children receiving oral cholera vaccine. To do so, we examined markers of enteropathy and micronutrients in the plasma and stool of 40 Bangladeshi children who received two doses of an OCV, Dukoral, and correlated those with immune responses to the vaccine.

## Methods and Materials

### Study subjects and sample collection

**Fig 1:**
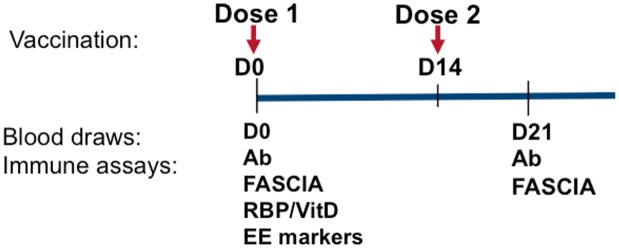
Time line for vaccination, blood draws and immunological assays. Ab = antibody measurements; FASCIA = flow cytometric assay of specific cell-mediated immune responses in activated whole blood; RBP = Retinol binding protein; VitD = 25-OH vitamin D; EE markers = biomarkers of environmental enteropathy.

As previously reported [12,19], we carried out this study in the urban slums of Mirpur, an area of Dhaka, Bangladesh. After obtaining written informed consent of the parents/ guardians as well as assent of the children wherever applicable, we enrolled children (age 3–14 years) that were healthy, did not have diarrhea in the preceding 14 days, and who were given two doses of a licensed killed whole cell oral cholera vaccine (OCV) Dukoral (WC-rBS), containing recombinant cholera toxin B subunit, 14 days apart. Individuals were recruited from February 22, 2011 to March 07, 2011. We obtained anthropometric measurements before vaccination and measured height for age (HAZ) Z-scores using WHO Multicenter Growth Reference Study Child Growth Standards; subjects were excluded if they had a Z score of less than -2. Subjects were also excluded if they had stool microscopy positive for parasites or had any febrile illness or antibiotic treatment in the past week. We obtained blood samples before vaccination (D0) and 7 days after the second dose of vaccination (D21, Fig 1**)**. We also collected stool samples before vaccination (D0). This study was approved by the Ethical and Research Review Committees of the icddr,b and the Institutional Review Board of Massachusetts General Hospital.

### Vibriocidal and *V*. *cholerae* antigen-specific plasma antibody levels

We determined vibrioicidal titers as previously described [15]. We quantified LPS- (prepared in house) [16] and cholera toxin B subunit- (CTB, gifts of A. M Svennerholm, *Göteborg* University) specific IgA, IgG and IgM antibody responses in plasma using a previously described kinetic enzyme-linked immunosorbent assay (ELISA) [14,17,18,19].

**T cell responses by FASCIA** (Flow cytometric Assay of Specific Cell-mediated Immune responses in Activated whole blood)

We performed FASCIA for evaluation of antigen stimulated lymphoblast subpopulations in blood, as previously described [12,19]. For *in vitro* antigenic stimulation, cholera holotoxin containing the G33D variant homopentameric B subunit (mCT, gifts of Randall K. Holmes, University of Colorado) [21] was used. After 6 days of culture, we separated the supernatant from the stimulated cells by centrifugation and added a protease inhibitor cocktail, followed by storing supernatants at -80°C for subsequent cytokine analysis by Luminex. To characterize the cell populations by surface expression of markers, we incubated them with various antibodies, including anti-CD3-phycoerythrin -Texas Red (Invitrogen, CA), anti-CD4-Amcyan, anti-CD45RA-V450, anti-integrin 7-PE, anti-CXCR5-AF488, anti-CCR7-PE-Cy7, and anti-CCR9-AF647 fluorochrome conjugated monoclonal antibodies (BD Bioscience, San Jose, CA). We used ammonium chloride (Sigma) solution to lyse red blood cells and the remaining lymphoblasts were resuspended in stabilizing fixative (BD Bioscience, San Jose, CA). We then acquired cell populations using a FACSAria III flowcytometer (BD Bioscience, San Jose, CA) and analyzed the data using the FACSDiva (BD Bioscience, CA) and the FlowJo software (TreeStar Inc., Oregon). Cellular proliferative responses are presented as the ratio (denoted as stimulation index, SI) of lymphoblast count with antigenic stimulation to the count without any stimulation. SI value greater than “1” indicates *V*. *cholerae* antigen-specific stimulation in compared to without a *V*. *cholerae* antigen or no stimulation.

### Evaluation of Cytokine level by Luminex

Stored FASCIA culture supernatants were analyzed for different cytokines as per manufacturers’ instructions using the Milliplex human cytokine/ chemokine kit (Millipore Corp., MA) and the Bio-Plex 200 system (Bio- Rad, Pennsylvania). We selected cytokines for analysis based on their importance in relation to infection, cell differentiation and relation to gut enteropathy [12,22].

### Biomarkers of enteropathy and nutritional measures

We performed ELISA to measure soluble CD14 (sCD14; R&D Systems, Minneapolis, MN, USA), endotoxin core IgG antibodies (EndoCAb; Hycult Biotech, Uden, Netherlands), and intestinal fatty acid binding protein (i-FABP; R&D Systems, Minneapolis, MN, USA) in plasma specimens [23]. All assays followed the instructions specified by the manufacturer. The samples were diluted at a ratio of 1:1000, 1:200 and 1:10 for sCD14, EndoCAb and i-FABP, respectively. We used flat-bottom MaxiSorp plates (Nunc) (Thermo # 442404) for the i-FABP assay. We quantified levels of MPO (Alpco, Salem, NH, USA; and Immundiagnostik, Bensheim, Germany) in fecal extracts as described in the package insert, using a dilution factor of 1:200. We assessed the concentration of AAT (ImmuChromGmBH, NC, USA) in fecal extracts, according to the package insert, using a dilution of 1:400 [7]. We measured retinol binding protein (RBP) and 25-OH vitamin D (VitD) by ELISA (R&D Systems and Roche, respectively) from plasma on day 0.

### Statistical analysis

We analyzed antibody responses as the fold change of titer from day 0 to 21. Memory T cell and cytokine responses were analyzed as the absolute change from day 0 to 21. For our comparison of EE markers between younger and older children, we conducted statistical comparisons using the Mann Whitney U test. To look at associations between immune responses and host factors, we conducted multiple linear regression analysis with Least Absolute Shrinkage and Selection Operator (LASSO) regularization to identify predictive host factors truly informative for each response of interest, and the final model for each response was determined based on the optimal tuning parameter using 10 fold cross-validation criteria. The LASSO method was used in this study since there was no high co-linearity among potential host factors. From the soft-threshold property of the LASSO in a linear model [24], the estimated regression coefficient is biased toward zero. To mitigate these bias problems, we reported a more unbiased estimation of the regression parameters from unpenalized multivariate linear regression using the selected factors in the LASSO. The age and gender covariates were added in all unpenalized multivariate linear regression models as default factors. All the LASSO analyses were performed using the "glmnet" package in R (www.r-project.org). The unpenalized multivariate linear regression was fitted using the function "lm" in R (www.r-project.org).

## Results

**Fig 2:**
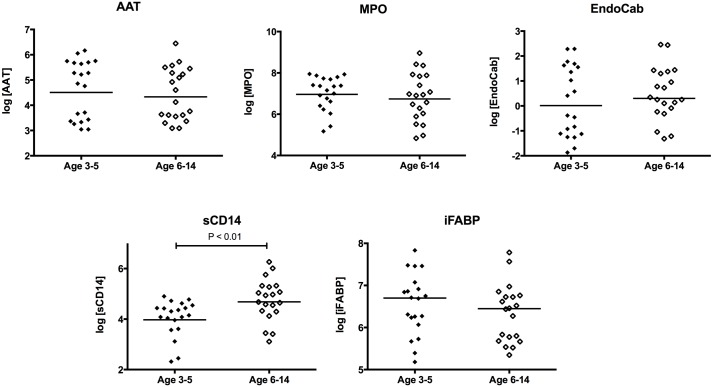
Comparison of enteropathy markers by age group. MPO = stool myeloperoxidase (ng/mL); AAT = stool alpha anti-trypsin (ug/mL); EndoCab = plasma endotoxin core antibody (GMU/mL); i-FABP = plasma intestinal fatty acid binding protein (pg/mL); sCD14 = plasma soluble CD14 (pg/mL).

We measured enteropathy markers and micronutrients in 40 children given 2 doses of a cholera toxin B subunit-containing oral cholera vaccine 14 days apart (Fig 1). There were 20 children ages 3–5 (“young children”) and 20 children ages 7–14 (“older children”). All the participants completed day 21 follow up. The characteristics of the cohort, by age grouping, are shown in Table 1. We did not find any age-specific differences in retinol binding protein or vitamin D levels. We also found comparable levels of enteropathy markers between young children and older children, with the exception of sCD14, which was higher in older children (Fig 2). We did not find any gender-specific differences in levels of EE markers.

**Table 1:** Host characteristics of 40 children who received 2 doses of an oral cholera vaccine.

Variable	Young children (n = 20)	Older children (n = 20)
Age, mean (yr, range)	4.8 (3–5)	10.5 (7–14)
Gender, females (%)	9 (45)	10 (50)
Blood type, O (%)	9 (45)	7 (35)
Height for Age, Z-score, mean (SD)	-0.08 (1.9)	-0.90 (2.1)
Plasma retinol binding protein, ug/mL, mean (SD)	6.4 (1.7)	6.3 (1.3)
Plasma Vitamin D, nmol/L, mean (SD)	38 (10)	37 (15)

We assessed the variance inflation factor and did not find high levels of co-linearity between host factors (VIF <4for all variables, range 1.18–1.78). We had previously reported *V*. *cholerae* antigen-specific immunological responses for this cohort of oral cholera vaccinees [12,19]. We conducted multivariate linear regression using selected factors from LASSO regularization, modeled with age and gender, to determine associations between immune responses and host factors, including micronutrient and EE markers. We showed the results of this analysis in Table 2, where all host factor(s) associated with each immunologic outcome with P < 0.10 are included. For each host factor, we determined an estimated effect as the changes in immunologic response with each one unit increase in the host factors. We found the LPS IgG response to be positively associated with plasma vitamin D (P = 0.02), i-FABP (P = 0.01), and sCD14 levels (P = 0.07). We found LPS IgA and CTB IgG responses to be positively associated with stool MPO (P = 0.02 and 0.03, respectively). We found that CT-specific effector memory T cell responses were positively associated with plasma i-FABP (P = 0.01) and negatively associated with plasma EndoCab (P = 0.02), and that the CCR9 gut-homing effector memory T cell subset had similar associations as the parent population. We found a negative association between IL-10 produced by T cells stimulated with CT, and stool AAT (P = 0.02).

**Table 2:** Host factors associated with immune responses to oral cholera vaccine in Bangladeshi children, determined by multivariate linear regression analysis with LASSO regularization.

Outcome	Associated host factors with P<0.10	Estimated Effect (Standard Error)	P-Value
Vibriocidal Antibody	None		
LPS IgG antibody	Vitamin D	0.02 (0.01)	0.02
	i-FABP	0.0005 (0.0002)	0.01
	sCD14	0.002 (0.001)	0.07
LPS IgA antibody	MPO	0.001 (0.0004)	0.02
CTB IgG antibody	MPO	0.0005 (0.0002)	0.03
CTB IgA antibody	None		
CT-specific Tem	EndoCab	-0.44 (0.19)	0.02
	i-FABP	0.003 (0.001)	0.01
CT-specific Tem FH	None		
CT-specific Tem expressing β7	None		
CT-specific Tem expressing CCR9	EndoCab	-0.56 (0.30)	0.06
	i-FABP	0.004 (0.002)	0.02
CT-specific IFN-γ response	None		
CT-specific IL-13 response	None		
CT-specific IL-17 response	None		
CT-specific IL-10 response	AAT	-1.31 (0.55)	0.02

mCT: G33D mutant cholera toxin; LPS: *V*. *cholerae* lipopolysaccharide; CTB: Cholera toxin B subunit; Tem: effector memory T cells; FH: follicular helper phenotype

## Discussion

Live oral vaccines including oral cholera vaccines fail to show similar levels of immunogenicity in developing countries as in developed countries [2,3,4]. Many factors such as maternal antibodies (placental or breast milk), infections at the time of vaccination, or gut enteropathy [2,6,8,25,26], are hypothesized to be the underlying causes of these decreased immune responses. However, the degree to which each factor influences immune responses to vaccines are yet to be elucidated. Among the factors, EE is prevalent in children of low-income countries, and we hypothesized that EE may be a cause of the lower immunogenicity of the oral cholera vaccine Dukoral in Bangladesh. However, our data did not support this hypothesis, but in contrast, suggested that environmental enteropathy (as evidenced by high enteropathy biomarkers) was positively associated with increased immunogenicity to vaccine antigens, particularly for antibody responses.

We examined several proposed biomarkers of enteropathy [5,7,27,28]. Intestinal Fatty Acid-Binding Protein 2 (i-FABP2) is mostly expressed in intestinal enterocytes [29], and studies have shown that i-FABP2 is an indicator of enterocyte damage and is associated with intestinal inflammation [30]. i-FABP2 is also involved in cell repair and proliferation [31,32] that may play crucial roles in immune responses to vaccines. A study of HIV enteropathy showed a concordant increase in i-FABP2 and duodenal helper T cells (CD4+) [28]. Here, we found that cholera toxin (CT) specific effector memory and gut homing memory T cell responses after vaccination were positively associated with i-FABP2. Similarly, MPO is known to be a specific marker of intestinal inflammation, and its concentration in stool has been associated with deficits in linear growth [33] [7]. We found that LPS IgA and CTB-IgG antibody responses to vaccination were positively associated with the concentration of MPO in stool.

Soluble CD14 (sCD14) is a glycoprotein that mediates the interaction of lipopolysaccharide (LPS, endotoxin) with antigen presenting cells such as macrophages to produce pro-inflammatory signalling in the presence of gram-negative bacteria [34,35,36]. sCD14 is expressed mainly by macrophages, and to a lesser extent by neutrophils [37]. In this study, we found a statistically non-significant association (P = 0.07) between *Vibrio cholerae* LPS-specific IgG antibody responses to vaccination and sCD14. Studies of oral vaccine responses in infancy have shown sCD14 to be both positively and negatively associated with oral polio vaccine antibody responses, which depended on the age of the child at which sCD14 was measured (6 weeks vs. 18 weeks of age) [38]. We hypothesize that inflammation at different ages may have differential effects on the outcome of the immune responses.

AAT is a protease inhibitor that crosses into the intestinal lumen as a result of increased gut permeability, and has been used as a marker of protein losing enteropathies [39]. High fecal AAT levels have also been associated with mucosal ulceration in acquired immunodeficiency syndrome (AIDS) patients [40]. Notable elevations of AAT are also seen in patients with shigellosis [41]. In this study, we found a negative association of CT specific, anti-inflammatory IL-10 responses with the level of AAT, consistent with the above findings that EE markers are associated with pro-inflammatory responses against vaccines. Campbell et al reported in a gut enteropathy study that the level of a regulatory cytokine (TGF-β) decreases with an increase in inflammatory cytokines [20].

While we saw a positive association between vitamin D levels and LPS antibody responses, we did not see any association between age, gender, blood group, or malnutrition (as assessed by HAZ score) and vaccine responses. While this exploratory study was not powered to detect such differences, our results suggest that enteropathy markers may be better predictors of vaccine responses than the markers of nutrition that were studied.

Lower immunity to enteric vaccines in developing countries in children is now well established through a number of studies, including ones examining oral cholera vaccine responses [2,3,4]. Among the factors hypothesized [2] to play a role in decreased immunity to OCV is gut enteropathy, which is commonly seen in resource-limited settings and believed to play a crucial role in underperformance of enteric vaccines. However, contrary to this hypothesis, we found a positive correlation of markers of gut enteropathy and immune responses to a cholera vaccine (Dukoral) in a cohort of Bangladeshi children 3–14 years of age. We hypothesize that the increased inflammation seen in enteropathy may facilitate a greater number of antigen presenting cell encounters with vaccine antigens, and that this is accompanied by increased B and T cell responses. However, more detailed studies with mucosal specimens and other enteric vaccines are needed to further our understanding of this observation.

The majority of published studies of the effect of enteropathy on vaccine responses have focused on infants [7,38]. This is one of the first studies to examine enteropathy markers in older children, and we postulate that enteropathy in older children may not have the same deleterious effects as those seen in infants. Notably, due to ethical and logistical limitations in infants, most of the enteropathy markers used in our study have only been histologically validated in adults.

The study has several limitations. Notably, we did not account for the intestinal microbiota, which has recently been shown to impact vaccine responses in infancy [42]. There may also be other unmeasured confounders that could impact immune responses. Additionally, we did not look at mucosal responses such as secretory IgA, nor at memory B cell responses. Lastly, we excluded severely malnourished children, and thus our findings may not be applicable to this population. Despite these limitations, we demonstrate here a positive association of enteropathy markers with immune responses to an oral vaccine. Further studies are warranted to delineate the mechanism through which this occurs.

## Supporting Information

S1 Checklist STROBE Checklist. (DOCX)
